# Heart Rate Measurement Based on 3D Central Difference Convolution with Attention Mechanism

**DOI:** 10.3390/s22020688

**Published:** 2022-01-17

**Authors:** Xinhua Liu, Wenqian Wei, Hailan Kuang, Xiaolin Ma

**Affiliations:** Hubei Key Laboratory of Broadband Wireless Communication and Sensor Networks, School of Information Engineering, Wuhan University of Technology, Wuhan 430070, China; liuxinhua@whut.edu.cn (X.L.); 252754@whut.edu.cn (W.W.); maxiaolin0615@whut.edu.cn (X.M.)

**Keywords:** heart rate measurement, region-of-interest, central difference convolution, attention mechanism

## Abstract

Remote photoplethysmography (rPPG) is a video-based non-contact heart rate measurement technology. It is a fact that most existing rPPG methods fail to deal with the spatiotemporal features of the video, which is significant for the extraction of the rPPG signal. In this paper, we propose a 3D central difference convolutional network (CDCA-rPPGNet) to measure heart rate, with an attention mechanism to combine spatial and temporal features. First, we crop and stitch the region of interest together through facial landmarks. Next, the high-quality regions of interest are fed to CDCA-rPPGNet based on a central difference convolution, which can enhance the spatiotemporal representation and capture rich relevant time contexts by collecting time difference information. In addition, we integrate the attention module into the neural network, aiming to strengthen the ability of the neural network to extract video channels and spatial features, so as to obtain more accurate rPPG signals. In summary, the three main contributions of this paper are as follows: (1) the proposed network base on central difference convolution could better capture the subtle color changes to recover the rPPG signals; (2) the proposed ROI extraction method provides high-quality input to the network; (3) the attention module is used to strengthen the ability of the network to extract features. Extensive experiments are conducted on two public datasets—the PURE dataset and the UBFC-rPPG dataset. In terms of the experiment results, our proposed method achieves 0.46 MAE (bpm), 0.90 RMSE (bpm) and 0.99 R value of Pearson’s correlation coefficient on the PURE dataset, and 0.60 MAE (bpm), 1.38 RMSE (bpm) and 0.99 R value of Pearson’s correlation coefficient on the UBFC dataset, which proves the effectiveness of our proposed approach.

## 1. Introduction

Heart rate is a vital indicator of health monitoring. Heart rate measurement is essential for health management, disease diagnosis and clinical research. Traditional contact heart rate measurement methods, including electrocardiograms, require specific equipment such as ECG technology. The surface electrode is in direct contact with the patient’s body surface, which brings inconvenience and discomfort to the patient, psychologically. In addition, ECG equipment is expensive, complicated to install, inconvenient to carry, and not suitable for real-time mobile heart rate monitoring. Remote photoplethysmography (rPPG) is a non-contact method to capture the periodic changes in skin color caused by the heartbeat through sensors such as cameras. The process of the method is as follows: (1) use the camera to capture the skin area (especially the face skin area) video; (2) analyze the periodic color changes in the skin area due to the blood flow pulsation caused by the heartbeat; (3) recover the corresponding rPPG signal and measure physiological indicators. The subtle color changes of skin in the video directly reflect changes in the rPPG signals, in other words, the deep learning models can capture the temporal variation of skin color to recover the rPPG signals. Today, severely affected by the COVID-19 pandemic, traditional heart rate measurement methods have greater safety risks. Close contact may cause infection, so that the study of non-contact rPPG signals measurement has attracted more attention [1,2,3].

With the continuous application of image video in the field of computer vision, AI has many applications in the field of healthcare such as HR measurement and blood pressure measurement [4], many non-contact heart rate measurement methods based on deep learning technology have begun to appear. Hsu et al. [5] proposed a method that used time-frequency representation to predict heart rate. The first step of their method was to detect the key points of the face and crop the region of interest, and then used the CHROM method to estimate the rPPG signals, and finally the representations were fed to VGG15 to estimate the heart rate. Špetlík et al. [6] proposed an end-to-end heart rate prediction model that included the extraction of the rPPG signals from the video sequence and the output of the predicted heart rate based on the rPPG signals received from the first stage. Niu et al. [7] aggregated the RGB signals in multiple regions of interest and converted them into spatial-temporal map representations, and then the spatial-temporal map representations were used to predict heart rate. Since the 2D convolution neural network only considers the spatial information of the video frame, many researchers began to use 3D convolution neural network to gain temporal information, which is significant for the rPPG signals recovery. Yu et al. [8] proposed PhysNet based on the spatiotemporal convolutional network, which can reconstruct precise rPPG signals from facial videos, and the final output of the model is the predicted rPPG signal. Tsou et al. [9] proposed Siamese-rPPG based on a Siamese 3D convolution network. Since different facial regions should reflect the same rPPG information, so they should be combined to improve the overall robustness for rPPG signals extraction. Lokendra et al. [10] proposed a novel denoising-rPPG network based on TCN architecture, which can model long sequences effectively. Moreover, Action Units (AUs) were used to denoise temporal signals by providing relevant information about the facial expression.

In order to extract more accurate rPPG signals, the attention mechanism has been widely used in the rPPG signals recovery [11,12]. Hu et al. [13] proposed a temporal attention mechanism for the extraction of the rPPG signals. The attention module strengthened the interaction capability of the previous and next frame information in the time dimension, which prevented abnormal changes in the temporal domain. Chen and McDuff [14] proposed an attention-based convolutional neural network to predict heart rate. The network combined an appearance model with a motion model; the attention mechanism was designed to direct the motion model to learn information more efficiently, the input of the motion model was normalized frame difference.

**Figure 1:**
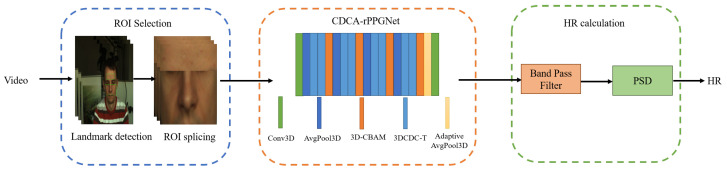
The overview of our framework.

In summary, the flow of the existing non-contact heart rate measurement methods mainly includes three steps: ROI selection, rPPG signal extraction, and heart rate measurement. The ROI selection is the first step to obtain the rPPG signal, which directly effects the quality of the rPPG signal [15]. There are some disadvantages in the existing ROI selection methods. A small number of skin pixels will lead to large quantized uncertainty [16]. Additionally, the down-sampling process of the skin pixels is found to deteriorate the quality of the rPPG. To learn spatiotemporal features effectively, we analyze the forehead and cheek independently, considering the fact that the absolute size of the forehead and cheek is larger than other facial regions and those regions contain rich rPPG information [17], which makes it easy for network to learn spatiotemporal features. In terms of the rPPG signal extraction, due to the fact that the conventional 3D convolutional neural network cannot extract spatiotemporal features effectively, since it is susceptible to irrelevant factors such as lighting changes, we proposed a central difference convolutional network (CDCA-rPPGNet) with an attention mechanism to obtain more accurate rPPG signal from the output of the ROI selection process. Figure 1 shows an overview of the method used to predict the heart rate. Our contributions are summarized as below:

1. We design the network based on central difference convolution to obtain rich time difference information for the extraction of the rPPG signals;
2. We propose a more reliable ROI extraction method. Face detection is used to extract the forehead and cheek, then we splice them as the input of the model;
3. The 3D-CBAM attention mechanism is designed to direct our network to learn information more efficiently and focus on more important features;
4. Experiments based on PURE [18] and UBFC [19] datasets demonstrate the robustness and effectiveness of our network.

## 2. Materials and Methods

### 2.1. ROI Selection

**Figure 2:**
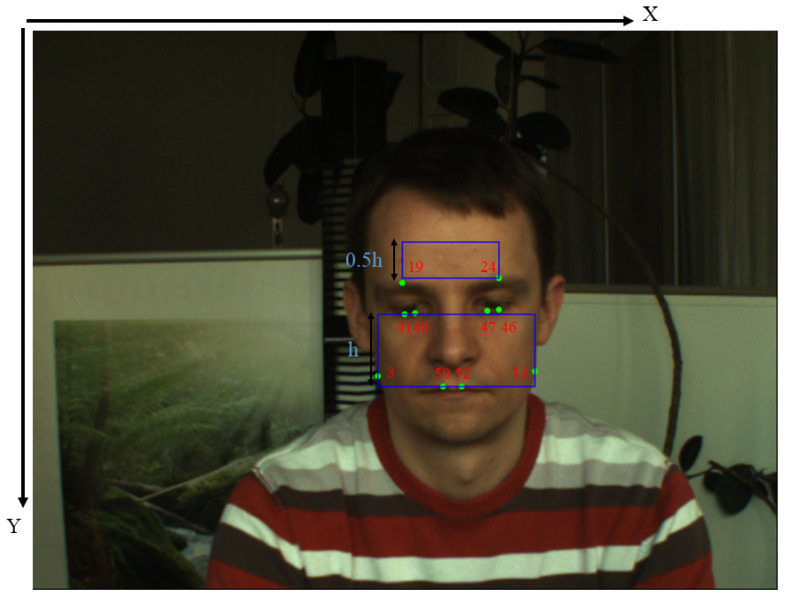
ROI selection.

All pixels of the face, except for the non-skin regions that contain no rPPG information, contribute to the rPPG signal. Most all heart rate estimation methods require the ROI selection. If we select the face region as the input of our model, the predicted rPPG signals will be interfered by the non-skin regions such as eyes and bread. Regarding only the cheek or forehead as input will ignore the other region that contains high-quality rPPG information, which will lead to a decrease in the robustness of the signal. In order to maximize the ratio of skin region, we splice the forehead and cheek as the input of our model. Face detection is used to extract the face region, precise facial landmarks are used to define the specific coordinates of the cropped regions. We use OpenFace [20] to get facial landmarks. For one thing, it offers high accuracy in face recognition; for another, it is easily integrated into today’s mobile devices, which means it does not require high computing power. To define the ROI, ten points of the 68 facial landmarks are used, the motivation is that we want to get high-quality ROI with the simplest operation, the selected ten landmarks can obtain all pixels of the cheeks as possible and make the forehead avoid the influence of hair. As shown in Figure 2, eight points of them are used to define the cheek. The other two points are used to define the forehead. The method of extracting cheek refers to [21]. In Equations (1) and (2), the coordinates of the ten points are applied to accurately define the cheek and forehead. The cheek and forehead are down-sampled to 64 × 96 pixels and 32 × 96 pixels respectively.

(1) $$\left\{\begin{array}{c} X_{cl}=X_{P_{3}}\\ Y_{cl}=\max\left(Y_{P_{40}},Y_{P_{41}},Y_{P_{46}},Y_{P_{47}}\right)\\ W_{crec}=X_{P_{13}}-X_{P_{3}}\\ H_{crec}=\min\left(Y_{P_{50}},Y_{P_{52}}\right)-Y_{cl} \end{array}\right.$$

(2) $$\left\{\begin{array}{c} X_{fl}=X_{P_{19}}\\ Y_{fl}=\min\left(Y_{P_{19}},Y_{P_{24}}\right)-0.5\times H_{crec}\\ W_{frec}=X_{P_{24}}-X_{P_{19}}\\ H_{frec}=0.5\times H_{crec}, \end{array}\right.$$

where $X_{*}$ and $Y_{*}$ denote the *x* and *y* coordinates of the top-left vertex respectively. $W_{*}$ is the width of ROI, $H_{*}$ is the height of ROI. As the ROI is extracted in this way, we maximize facial pixels as the input of our network, which can weaken the impact of background and head movements as much as possible.

### 2.2. Central Difference Convolution

The process of extracting the rPPG signal is to obtain the temporal variation of skin color. In order to extract the spatiotemporal features more effectively, Yu et al. [22] first applied central difference convolution for the task of gesture recognition, which is beneficial for the rPPG signal recovery by better capturing time difference information [23]. Central difference convolution is developed based on conventional 3D convolution and is the basic unit of our network for heart rate measurement. Two steps are included in the operation process of traditional 3D convolution: (1) sampling the local receptive field C on the input feature map X; (2) aggregation of sampled values via weighted summation. Compared with conventional 3D convolution, temporal central difference convolution (3DCDC-T) enhances the spatiotemporal representation through considering temporal central differences. It captures numerous temporal contexts, which is suitable for heart rate measurement. The sample local receptive field C is divided into two types of regions: (1) the current time step $R^{\prime }$; (2) the adjacent time steps $R^{\prime \prime }$. Temporal central difference convolution also contains two steps similar to conventional 3D convolution; the output of 3DCDC-T could be calculated by the following Equation (3).

(3) $$\begin{array}{cc} 3\mathrm{DCD}C_{T}\left(p_{0}\right)\;= & \sum_{p_{n}\in C}\omega \left(p_{n}\right)\cdot x\left(p_{0}+p_{n}\right)\\ \; & +\theta \cdot \left(-x\left(p_{0}\right)\cdot \sum \omega \left(p_{n}\right)\right),\\ \; & \;\;\;p_{n}\in R^{\prime \prime } \end{array}$$

where $p_{0}$ represents the current position on both input and output feature maps while $p_{n}$ denotes the position in the local receptive field C. Hyperparameter θ∈[0,1] tradeoffs the contribution between intensity-level and gradient-level information. 3DCDC-T is adopted in CDCA-rPPGNet for rPPG signal extraction.

### 2.3. 3D Convolutional Block Attention Module

**Figure 3:**
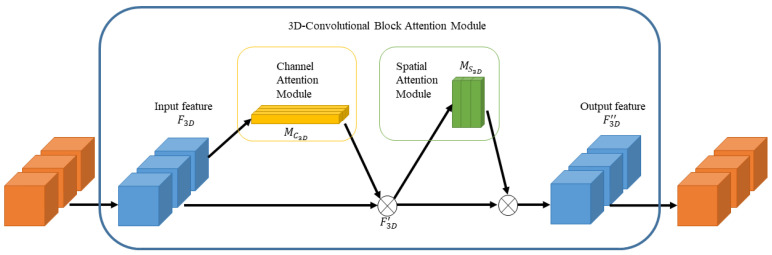
The structure of 3D-CBAM.

CBAM [24] attention mechanism is a lightweight and effective attention module that can be directly applied to convolutional neural networks. For feature maps generated by convolutional neural networks, CBAM calculates two dimensions of attention weights: channel and spatial, and then the corresponding elements of the attention map and the feature map are multiplied for adaptive feature refinement. We extended CBAM from 2D to 3D, its structure is shown in Figure 3. The channel attention module focuses on the feature channels that are decisive for the extraction of rPPG signals.

**Figure 4:**
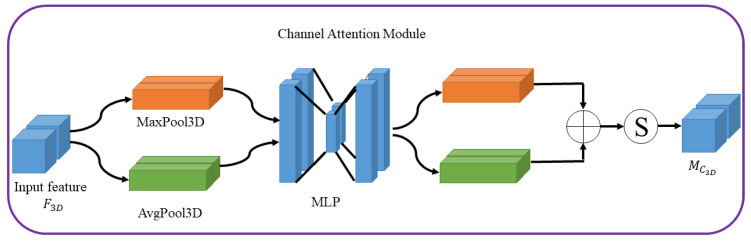
Diagram of the channel attention module.

As shown in Figure 4, the diagram of channel attention, the feature map $F_{3D}$ is processed by channel attention module to 1D channel attention map $M_{C_{3D}}$, which is multiplied by $M_{C_{3D}}$ to get $F_{3D}^{\prime }$.

The output $F_{3D}^{\prime }$ can be obtained by the following formula:

(4) $$\begin{array}{c} M_{C_{3D}}=\sigma \left(MLP\left(\mathrm{AvgPool}3D\left(F_{3D}\right)\right)+MLP\left(\mathrm{MaxPool}3D\left(F_{3D}\right)\right)\right)\\ =\sigma \left(W_{1}\left(W_{0}\left(F_{\mathrm{avg}\;}^{C}\right)\right)\right)+\sigma \left(W_{1}\left(W_{0}\left(F_{\max}^{C}\right)\right)\right) \end{array}$$

(5) $$F_{3D}^{\prime }=F_{3D}⊗M_{C_{3D}},$$

where σ represents the sigmoid function, AvgPool3D and MaxPool3D represent the average-pooling and maximum-pooling operations. *MLP* represents the multi-layer perceptron, the weights $W_{1}$ and $W_{0}$ are shared for both inputs. The symbol ⊗ represents the element-wise multiplication.

**Figure 5:**
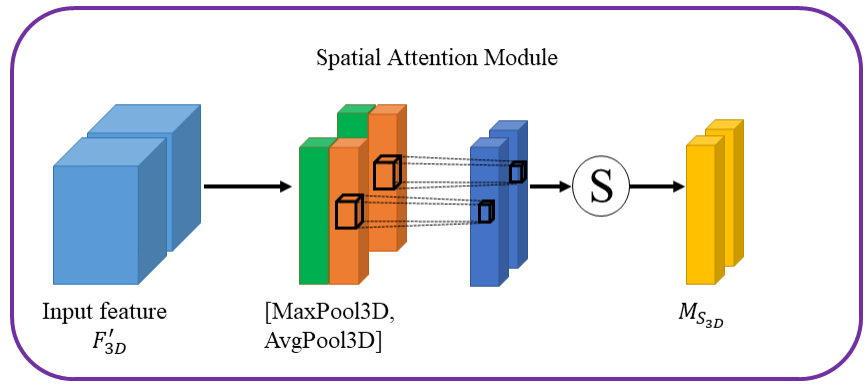
Diagram of the spatial attention module.

Diagram of spatial attention is shown in Figure 5, the feature $F_{3D}^{\prime \prime }$ is processed by the spatial attention module, spatial attention module focuses on which pixels in the RGB image sequence have a greater contribution to the extraction of rPPG signal.

Hence, the output feature map $F_{3D}^{\prime \prime }$ can be calculated by:

(6) $$M_{S_{3D}}=\sigma \left(f^{7\times 7\times 7}\left(\left[\mathrm{AvgPool}3D\left(F_{3D}^{\prime }\right),\mathrm{Max}\mathrm{Pool}3D\left(F_{3D}^{\prime }\right)\right]\right)\right)$$

(7) $$F_{3D}^{\prime \prime }=F_{3D}^{\prime }⊗M_{S_{3D}},$$

where σ represents the sigmoid function and $f^{7\times 7\times 7}$ denotes a 3D convolution layer with the filter size of 7 × 7 × 7. AvgPool3D and MaxPool3D represent the average-pooling and maximum-pooling operations. The symbol ⊗ represents the element-wise multiplication.

### 2.4. Network Architecture

**Figure 6:**
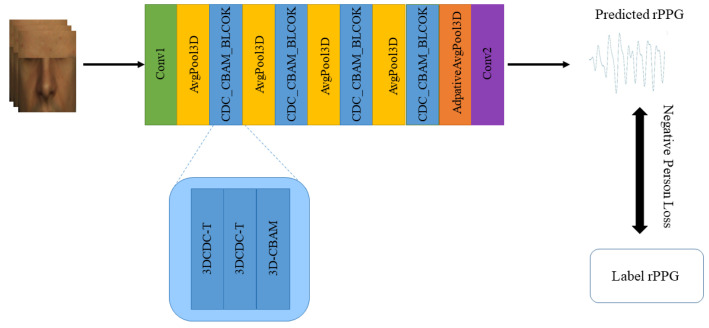
The overview of CDCA-rPPGNet.

To efficiently predict the rPPG signal, we propose an efficient network. An overview of CDCA-rPPGNet is presented in Figure 6. The first convolution layer aims to learn multiple combinations of color for more effective rPPG information. CDC_CBAM_BLOCK consists of two 3DCDC-T and 3D-CBAM, which is adopted to extract the rPPG information in the spatiotemporal domain. It helps to learn more effective temporal contexts and is less disturbed by non-skin pixels. The last layer aims to aggregate channels for final rPPG signals. AvgPool and AdpativeAvgPool are used to reduce the feature map size, which can weaken the impact of facial motion. The structure of CDCA-rPPGNet, including approximately 0.66 M parameters, is described in Table 1.

### 2.5. Loss Function

Our proposed network architecture is developed to recover rPPG signals with similarity in trend and to accurately estimate pulse peak time positions that match with ground truth rPPG signals. A suitable loss function needs to be designed to guide our networks. The frequently used loss functions are inappropriate for rPPG signals, since both rPPG signals (from facial video) and PPG signals (from contact measurement) reflect the blood volume changes, but their exact values are not the same. We only care about the trend of signals but ignore the specific value, so Negative Pearson Correlation is used as the loss function. Pearson Correlation indicates the linear similarity between rPPG signals and PPG signals, which can guide our networks to maximize the trend similarity. It is formulated as:

(8) $$\;\mathrm{Loss}\;=1-\frac{\sum_{i=1}^{T}\left(x\left(i\right)-\bar{x}\right)\left(y\left(i\right)-\bar{y}\right)}{\sqrt{\sum_{i=1}^{T}{\left(x\left(i\right)-\bar{x}\right)}^{2}}\sqrt{\sum_{i=1}^{T}{\left(y\left(i\right)-\bar{y}\right)}^{2}}},$$

where *x* is the predicted rPPG signals, *y* donates the ground truth rPPG signals, and *T* is the length of the rPPG signals. $\bar{x}$ and $\bar{y}$ denote the average values of two signals respectively. The Pearson Correlation coefficient, ranged from −1 to +1, indicates the similarity between two signals. The correlation of −1 represents a negative correlation between two signals, the value 0 represents no linear correlation. The value +1 represents a positive correlation between two signals. Our goal is that the predicted rPPG signals should be strongly correlated with the ground truth rPPG signals.

## 3. Results

To train and evaluate our network efficiently, experiments based on PURE and UBFC datasets were conducted. We used three performance metrics for heart rate measurement: mean absolute error (MAE), root mean squared error (RMSE), Pearson’s correlation coefficient (R).

### 3.1. Datasets

PURE: The dataset contains ten subjects, every subject contains six different activities (steady, talking, slow head translation, fast head translation, small head rotation, medium head rotation). It is a fact that talking and head movements will cause large light variation, which makes it difficult to recover rPPG signals. In a total of 60 videos, every video is about 1 min and all videos were recorded by the industrial camera at 30 fps with 640 × 480 pixels spatial resolution. The ground truth PPG signals were captured with a finger pulse oximeter pulox CMS50E with a sampling rate of 60 Hz.

UBFC-rPPG: the dataset includes 42 videos of 42 subjects; each subject has one video, every video is about one minute. In the video recording process, in order to make the subject’s heart rate change, the subject is asked to play a game that can trigger the heart rate change. The video was recorded by Logitech C920 HD Pro at 30 fps with a spatial resolution of 640 × 480 pixels. A finger pulse oximeter CMS50E was used to capture the ground truth rPPG signals with a 60 Hz sampling rate. Since UBFC-rPPG dataset is really small, we performed data augmentation on the sample, we flipped each sample left and right, which doubles the number of samples.

**Figure 7:**
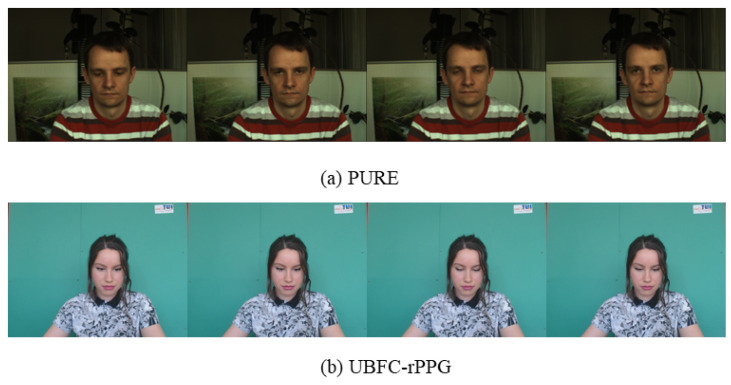
Examples of PURE and UBFC datasets.

The examples of the two datasets are shown in Figure 7. For the PURE dataset, the training set contains six subjects (36 videos of six subjects) and the testing set contains the other four subjects (24 videos of four subjects). For the UBFC dataset, the training set contains 26 subjects (26 videos of 26 subjects) and the test set contains 16 subjects (16 videos of 16 subjects). Since the UBFC-rPPG dataset is extremely small, we performed data augmentation on the sample by flipping each sample left and right, which doubles the sample size.

### 3.2. Evalution Metrics

At present, three performance metrics are used for heart rate measurement: mean absolute error (MAE), root mean squared error (RMSE), Pearson’s correlation coefficient (R).

1. Mean absolute error (MAE)
  MAE is the average value of the absolute deviations of all estimated HR and the ground truth HR. It can be expressed as:
  (9) $$HR_{\mathrm{mae}\;}=\frac{1}{n}\sum_{i=1}^{n}\left|HR_{predict}^{\left(i\right)}-HR_{gt}^{\left(i\right)}\right|.$$
2. Root square mean error (RMSE)
  RMSE is the average value of the standard deviations of all estimated HR and the ground truth HR. It can be calculated by the following formula:
  (10) $$HR_{rmse}=\sqrt{\frac{1}{n}\sum_{i=1}^{n}{\left(HR_{\mathrm{predict}\;}^{\left(i\right)}-HR_{gt}^{\left(i\right)}\right)}^{2}}.$$
3. Pearson’s Correlation Coefficient (R)
  R measures the linear dependence between two signals.
  (11) $$HR_{R}=\frac{\mathrm{Cov}\left(HR_{\mathrm{predict}},HR_{gt}\right)}{\sqrt{\mathrm{Cov}\left(HR_{\mathrm{predict}},HR_{\mathrm{predict}}\right)}\sqrt{\mathrm{Cov}\left(HR_{gt},HR_{gt}\right)}},$$

where $HR_{\mathrm{predict}\;}$ and $HR_{\mathrm{gt}\;}$ denote the estimated HR and the ground truth HR respectively, and *N* is the number of heart rate samples. Cov(x,y) denotes the covariance of *x* and *y*.

### 3.3. Parameters Setting

For our experiments, due to the different frequencies of the ground truth rPPG signals and the video frame sampling frequency, we should normalize the rPPG signal first then subsample it to the video frame. The input of the model is the continuous ROI images $x\in R^{128\times 96\times 96\times 3}$, ROI images were generated by the method mentioned in Section 2.1. To increase the sample size, we sample in steps of eight frames in the two datasets. The predicted rPPG signal was filtered by a sixth-order Butterworth bandpass filter, which was applied with a frequency between 0.7 to 2.5 Hz. The HR is estimated by the power spectral density (PSD) analysis from the filtered signals. We used the windows size of 10 s and step size of 2 s to calculate HR.

We used the Adam optimizer to train our model, the batch size is eight, the learning rate is set to 0.0002, the model is trained with 30 epochs. ReLU activation is used in each convolutional layer. All network components are implemented by Pytorch framework and trained with Quadro P6000.

### 3.4. Ablation Study

To evaluate the effectiveness of our model for non-contact heart rate measurement, we conduct experiments on the PURE dataset and the UBFC dataset. We perform the following ablation study: (1) replace 3DCDC-T with conventional 3D convolution and remove the attention module; (2) only remove the attention module. Some traditional methods and deep learning methods are used for comparison, the result shows that the proposed method outperforms other methods.

The experimental results of the PURE dataset are shown in Table 2. The results obtained by deep learning methods are generally better than traditional methods, and our proposed method achieved the best result. Existing deep learning models cannot capture rich temporal contexts well. In our model, we use 3DCDC-T to reduce the influence of noise. Besides, 3D-CBAM is used to help our network to learn more important features, which can improve the effectiveness of our method. The decrease of MAE and RMSE indicates that 3DCDC-T and the attention module are effective for recovering the rPPG signals, and the best result is achieved by combining them together.

We also evaluate the proposed method on UBFC dataset, and the results are shown in Table 3. Our proposed method achieves 0.60 MAE (bpm), 1.38 RMSE (bpm) and 0.99 R value of Pearson’s correlation coefficient. Compared with existing deep learning methods, our proposed method outperforms the other deep learning baseline. Same as the result obtained on PURE dataset, 3DCDC-T and 3D-CBAM attention module are also helpful for the extraction of the rPPG signals on UBFC dataset.

**Figure 8:**
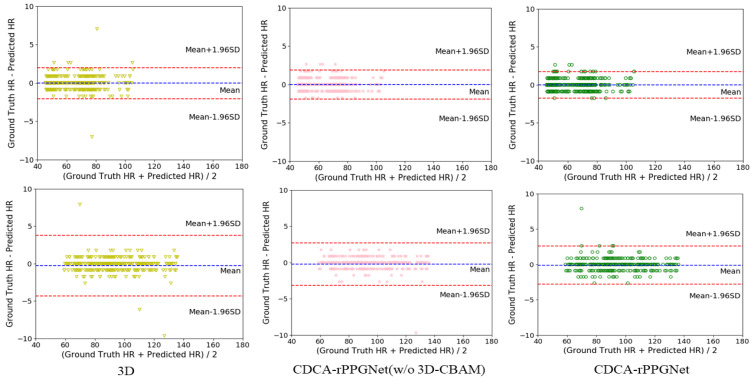
Bland–Altman plots between the estimated HR and the ground truth HR on PURE dataset (**top**) and UBFC dataset (**bottom**).

In addition, as shown in Figure 8, we analyze the influence of 3DCDC-T and the attention module via the Bland–Altman plot. The estimated HR range is within the ground truth HR. It can be analyzed through the Bland–Altman that the HR distribution is more consistent with the addition of 3DCDC-T and the attention module. In conclusion, the plots visually indicate that our proposed method is more effective and robust.

**Figure 9:**
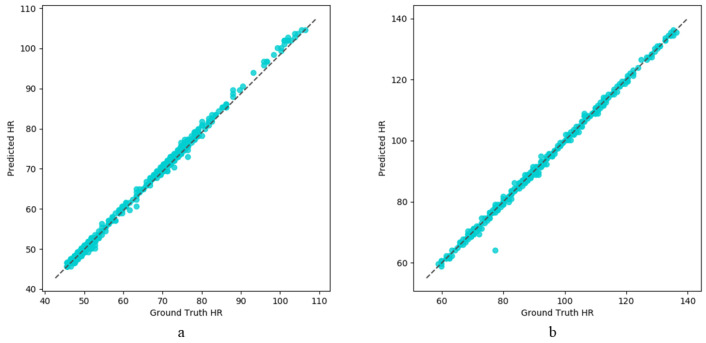
(**a**) scatter plot between the ground truth HR and the estimated HR on the PURE dataset; (**b**) scatter plot between the ground truth HR and the estimated HR on the UBFC dataset.

To show the results of our model more intuitively, we analyze the linear similarity of two signals via the scatter plot. As shown in Figure 9, these scatter plots indicate that the linear correlation of the HR predicted by our model and the ground truth HR is very strong.

**Figure 10:**
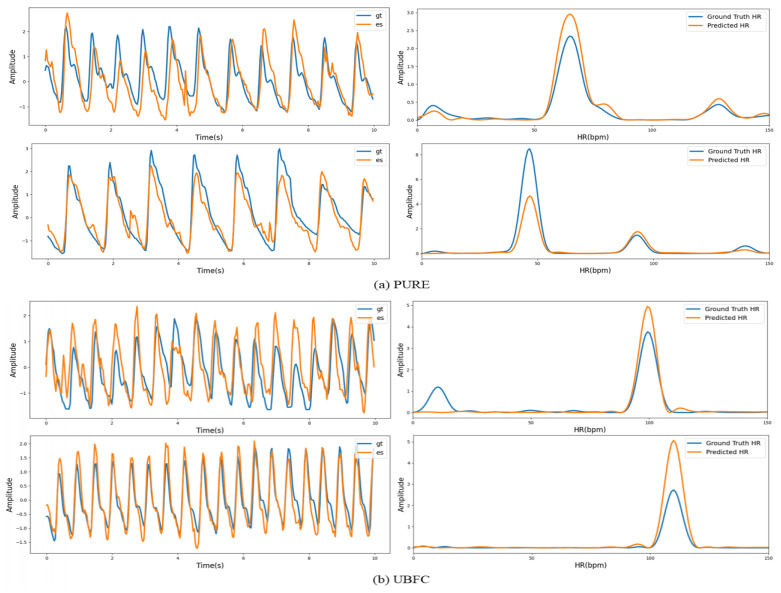
(**a**) Visualization of the predicted rPPG signals in the PURE dataset. (**b**) Visualization of the predicted rPPG signals in the UBFC dataset.

Figure 10 visualizes some examples of the estimated rPPG signals and corresponding power spectrum. It can be seen intuitively that the predicted signal and the ground truth rPPG signals almost have the same trend, which proves that our model is effective for remote heart rate measurement.

## 4. Discussion

We proposed a central difference convolution network with an attention mechanism to recover rPPG signals from facial video. We evaluated the proposed method on two public datasets. The experimental results indicate that our proposed method was more accurate than the previous method. The proposed method mainly included two steps: ROI selection, rPPG signals prediction using our model.

First of all, the ROI selection is very important to the recovery of the rPPG signals, because the region of the face in the video is originally small, which means that the skin pixels can be used to predict rPPG signals are really scarce. If the video is compressed, the quality of the skin pixels will be worse, which will make it difficult to estimate the rPPG signals. In theory, all skin pixels of the face have an effect on the extraction of signals, but the existing methods are difficult to use skin pixels efficiently. To solve this problem, we take the two ROIs that have the largest area and the most rPPG information as the input of our model. The cheek and forehead are affected by the background environment and light to varying degrees, but they both reflect the rPPG information. Using both of them can reduce the impact of the background on our signal extraction, which makes the rPPG signals we extract more robust. At the same time, we minimize the learning difficulty of our network, which can make the network more focused on learning useful information to recover the rPPG signals.

The next step is the construction of the neural network. The rPPG signals are essentially a kind of time series, the signals change with time. 3DCDC-T has the ability to better obtain the differences in time context, which is useful for the extraction of the rPPG signal, so we use 3DCDC-T to extract features. Changes in the rPPG signals are reflected from the subtle changes in the color of skin; they are relatively shallow features. Unlike video classification or action recognition, our task does not require a deep network to extract [33]. Therefore, we proposed a lightweight network. The attention mechanism is used to learn more important features for signals recovery. Some pixels on the cheek or forehead, such as bangs, do not contribute exactly to the rPPG signals recovery. So the attention mechanism can be added, which can guide our model to learn regions and channel features that are more essential.

In conclusion, the ROI selection is as important as the signals extraction method. Two steps of our method have an impact on heart rate prediction. Our proposed method showed an MAE of 0.46 bpm in the PURE dataset by combining two stages. We also achieved an MAE of 0.60 bpm in the UBFC dataset. Although our proposed method performed well in experiments, there are still some limitations. First of all, our preprocessing process requires accurate face detection and landmarks, which will not work normally if the subject’s face is partially occluded or the subject is in motion. In addition, a deep learning based method requires a large number of training samples, which is a challenge for remote heart rate measurement. Since heart rate distribution is between 40 bpm and 150 bpm for most samples, our method fails to achieve good accuracy for predicting abnormal heart rate values. Although there are still some limitations, our proposed method has the potential to make a contribution to the practical use of assisted living, which is suitable for our measurement scenarios.

## 5. Conclusions

Remote HR measurement plays an important role in the field of healthcare. Due to the COVID-19 pandemic, remote HR measurement may be widely used in disease diagnosis and real-time heart rate monitoring. However, the process of most existing rPPG methods is too complicated to be applied to real scenarios. In this paper, we proposed a central differential convolutional network with an attention mechanism for more robust rPPG signal measurement from facial video. The preprocessing part uses face key point detection to segment and splice the regions of interest of the face. Compared with the conventional 3D convolution, the improved 3DCDC-T can estimate the rPPG signal more accurately by enhancing the spatiotemporal representation with an abundant temporal background. The attention mechanism can guide the network to learn more critical feature channels and spatial features for the rPPG signal recovery. On the one hand, our network only includes approximately 0.66 M parameters, which means we can easily deploy the model on the mobile device and, on the other hand, experimental results on two public datasets—PURE and UBFC-rPPG—demonstrate the effectiveness of our proposed method. Our model achieves an MAE of 0.46 bpm and an MAE of 0.60 bpm on the PURE dataset and the UBFC dataset, respectively, which is superior to other current methods. In the future, we will be looking to improve the robustness of the model in low-constraint environments, such as head movements, and reduce the impact of unbalanced HR distribution.

## Figures and Tables

**Table 1:** The detailed structure of CDCA-rPPGNet.

Input Size	Layer	Stride	Kernel Size	Output Size
128 × 96 × 96 × 3	Conv 2D	1 × 1 × 1	1 × 5 × 5	128 × 96 × 96 × 16
128 × 96 × 96 × 16	AvgPool3D	1 × 2 × 2		128 × 48 × 48 × 16
128 × 48 × 48 × 16	CDC_CBAM_Block	1 × 1 × 1	3 × 3 × 3	128 × 48 × 48 × 32
128 × 48 × 48 × 32	AvgPool3D	1 × 2 × 2		128 × 24 × 24 × 32
128 × 24 × 24 × 32	CDC_CBAM_Block	1 × 1 × 1	3 × 3 × 3	128 × 24 × 24 × 64
128 × 24 × 24 × 64	AvgPool3D	1 × 2 × 2		128 × 12 × 12 × 64
128 × 12 × 12 × 64	CDC_CBAM_Block	1 × 1 × 1	3 × 3 × 3	128 × 12 × 12 × 64
128 × 12 × 12 × 64	AvgPool3D	1 × 2 × 2		128 × 6 × 6 × 64
128 × 6 × 6 × 64	CDC_CBAM_Block	1 × 1 × 1	3 × 3 × 3	128 × 6 × 6 × 64
128 × 6 × 6 × 64	AdaptiveAvgPool3D			128 × 1 × 1 × 64
128 × 1 × 1 × 64	Conv 1D	1 × 1 × 1	1 × 1 × 1	128 × 1 × 1 × 1

**Table 2:** HR Estimation Results on the PURE dataset.

Methods	MAE	RMSE	R
CHROM [25]	2.07	2.50	0.99
2SR [26]	2.44	3.06	0.98
LiCVPR [27]	28.22	30.96	−0.38
POS [28]	3.14	10.57	0.95
HR-CNN [6]	1.84	2.37	0.98
Physnet [8]	1.90	3.44	0.98
Deephys [14]	0.83	1.54	0.99
3D	0.91	1.34	0.99
CDCA-rPPGNet (w/o 3D-CBAM)	0.65	1.12	0.99
CDCA-rPPGNet	0.46	0.90	0.99

**Table 3:** HR Estimation Results on the UBFC dataset.

Methods	MAE	RMSE	R
CHROM [25]	3.44	4.61	0.97
POS [28]	2.44	6.61	0.94
Meta-rPPG [29]	5.97	7.42	0.53
CK [30]	2.30	3.80	0.98
HeartTrack [31]	2.41	3.37	0.98
PulseGAN [32]	1.19	2.10	0.98
3D	0.75	2.24	0.99
CDCA-rPPGNet (w/o 3D-CBAM)	0.69	1.73	0.99
CDCA-rPPGNet	0.60	1.38	0.99
